# Investigation on Species Authenticity for Herbal Products of *Celastrus Orbiculatus* and *Tripterygum Wilfordii* from Markets Using ITS2 Barcoding

**DOI:** 10.3390/molecules23040967

**Published:** 2018-04-21

**Authors:** Jingjing Zhang, Xin Hu, Ping Wang, Bisheng Huang, Wei Sun, Chao Xiong, Zhigang Hu, Shilin Chen

**Affiliations:** 1 College of Pharmacy, Hubei University of Chinese Medicine, No. 1 Huangjiahu West Road, Hongshan District, Wuhan 430065, China; zhangjingjing851@126.com (J.Z.); xinhu0624@163.com (X.H.); pwang54@yahoo.com.cn (P.W.); hbsh1963@163.com (B.H.); xiongchao080190@126.com (C.X.); 2 Zhang Zhongjing Traditional Chinese Medicine College, Henan Key Laboratory of Zhang Zhongjing Formulae and Herbs for Immunoregulation, Nanyang Institute of Technology, No. 80 Changjiang Road, Wancheng District, Nanyang 473004, China; 3 Institute of Chinese Materia Medica, China Academy of Chinese Medicinal Sciences, No.16 Dongzhimenneinanxiaojie street, Dongcheng District, Beijing 100700, China; wsun@icmm.ac.cn; 4 Zhan Yahua National Famous Traditional Chinese Medicine Experts Inheritance Studio, No. 1 Huangjiahu West Road, Hongshan District, Wuhan 430065, China

**Keywords:** ITS2, DNA barcoding, *Celastrus orbiculatus*, *Tripterygium wilfordii*, traceability

## Abstract

Herbal material is both a medicine and a commodity. Accurate identification of herbal materials is necessary to ensure the safety and effectiveness of medication. With this work, we initiated an identification method to investigate the species authenticity for herbal products of *Celastrus orbiculatus* and *Tripterygum wilfordii* utilizing DNA barcoding technology. An ITS2 (internal transcribed spacer two) barcode database including 59 sequences was successfully established to estimate the reliability of species-level identification for *Celastrus* and *Tripterygium*. Our findings showed that ITS2 can effectively and clearly distinguish *C. orbiculatus*, *T. wilfordii* and its congeners. Then, we investigated the proportions and varieties of adulterant species in the herbal markets. The data from ITS2 region indicated that 13 (62%) of the 21 samples labeled as “Nan-she-teng” and eight (31%) of the 26 samples labeled as “Lei-gong-teng” were authentic; the remaining were adulterants. Of the 47 herbal products, approximately 55% of the product identity were not in accordance with the label. In summary, we support the efficacy of the ITS2 barcode for the traceability of *C. orbiculatus* and *T. wilfordii*, and the present study provides one method and reference for the identification of the herbal materials and adulterants in the medicinal markets.

## 1. Introduction

**Figure 1:**
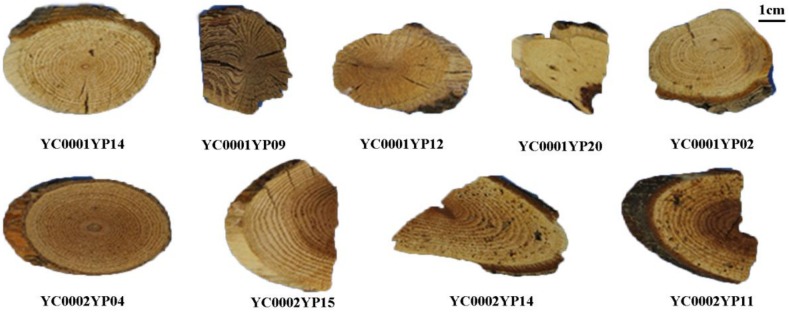
Representative herbal decoction samples available from markets in different Chinese provinces. Voucher No.: YC0001YP14, YC0001YP09, YC0001YP12, YC0001YP20, and YC0001YP02 were labeled as Nst; YC0002YP04, YC0002YP15, YC0002YP14, and YC0002YP11 were labeled as Lgt.

*Tripterygium wilfordii* Hook F is a perennial woody vine distributed in southeast China and East Asia [1]. Lei-gong-teng (Lgt) is the Chinese vernacular name for *T. wilfordii*, which is considered as an alternative or complementary therapy for the treatment of autoimmune and inflammatory diseases, including rheumatoid arthritis (RA), systemic lupus erythematosus, ankylosing spondylitis, psoriasis, and idiopathic IgA nephropathy [2,3]. However, because its active ingredient is also toxic, it inevitably produces various toxic and side effects, such as hepatotoxicity, nephrotoxicity, blood system toxicity, etc., and its toxic side effects cannot be ignored [4,5]. *Celastrus orbiculatus* Thunb. is another plant of the same family with *T. wilfordii* (Celastraceae). Nan-she-teng (Nst) is the vernacular name for *C. orbiculatus* and it is widely distributed in native. The extracts and some compounds isolated from *C. orbiculatus* were reported to have anti-tumor, anti-inflammatory, analgesic, antibacterial, antiviral, anti-fertility, and other pharmacological activities with low adverse effects in vitro and in vivo [6,7]. Still, *C. orbiculatus* is easily confused with *T. wilfordii* or other closely related plants due to morphological similarities, and many of them are frequently misused as Lgt or Nst. Our observations also found this to be the case (Figure 1). Nevertheless, traditional plant identification methods, like morphological, microscopic, and chemical identification, have some limitations for species identification [8]. Therefore, the identification of these species becomes a difficult task, and products can be easily adulterated with substitutes. A clear need exists for the development of an effective and rapid identification method for *C. orbiculatus* and *T. wilfordii* to ensure the safety of medication.

DNA barcoding, a relatively new technology that complements taxonomy, has significantly facilitated the identification and description of new species, especially when morphological characteristics of a specimen are absent [9,10]. This technique has become popular and widely used in medicine, ecology, food, and other fields for the identification of materials [11,12,13,14,15]. Many single or multiple regions in nuclear and plastid genomes have been screened as DNA barcodes for plants, animals and fungi including cytochrome c oxidase subunit 1 (CO1), internal transcribed spacer (ITS), *psbA-trnH*, *matK*, *rbcL*, and *rpoC1* [16,17]. Among barcode markers, ITS2 has gained popularity with researchers due to its high variability and power to determine species, and also has been proposed as a standard DNA barcode for medicinal plant authentication [18]. Many reports have validated the reliability of ITS2 for authenticating medicinal plants and their adulterants [19,20,21,22]. However, molecular identification based on the ITS2 barcode for *C. orbiculatus* and *T. wilfordii* have yet to be conducted.

Our specific objectives were as follow: 1. Estimate the reliability of using ITS2 for the identification of *C. orbiculatus*, *T. wilfordii* and other closely related species. 2. Examine the species authenticity of commercially available Nst and Lgt using established ITS2 barcoding technique and set up a database. The present study indicates that DNA barcoding is a convenient tool to implement for identification and market supervision.

## 2. Results

### 2.1. ITS2 Barcode Database for C. orbiculatus, T. wilfordii, and Other Closely Related Species

First, an ITS2 barcode database for *C. orbiculatus*, *T. wilfordii* and other closely related species was established to ensure efficiency of the molecular identification. Thirty-eight plant samples, representing *T. wilfordii* and nine *Celastrus* species, were collected from different localities in China and were used to establish the ITS2 database. These samples were all identified by taxonomic experts according to the Flora of China (http://foc.e ora.cn/). Using ITS2 barcode technology [19,23], PCR (polymerase chain reaction) amplifications were successful for all plant materials. To extend our ITS2 database and cover more genotypes, 21 reference ITS2 sequences were downloaded from GenBank. On the whole, the ITS2 barcode database contains 50 ITS2 sequences for *Celastrus* sp. (from which 11 for *C. orbiculatus*) and nine sequences from *T. wilfordii* (Table 1 and Table S1).

**Figure 2:**
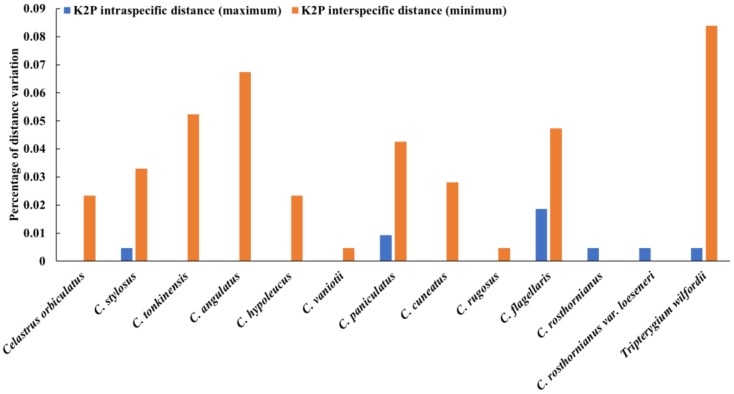
The histogram of inter/intra-specific divergence of the ITS2 barcode for *C. orbiculatus*, *T. wilfordii,* and other closely related species.

The results showed that the mean sequence length of ITS2 for the 12 *Celastrus* species and *T. wilfordii* varies from 221 bp to 231 bp, the GC (Guanine and Cytosine) contents range from 59.6% to 68.6%, the number of haplotypes ranged from one to three and the number of variable sites ranged from zero to four (Table 1). Except for *C. rosthornianus* and *C. rosthornianus var. loeseneri*, the minimum interspecific Kimura 2-parameter (K2P) distance was larger than the maximum intraspecific distance (Figure 2).

### 2.2. Identification of Species-Level for C. orbiculatus, T. wilfordii, and Other Closely Related Plants

**Figure 3:**
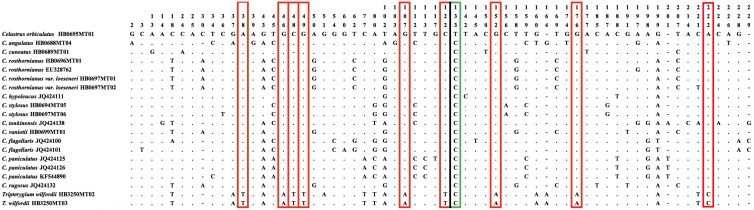
Single nucleotide polymorphisms (SNPs) from the ITS2 sequences of *Celastrus* species and *T. wilfordii*. All 13 species in the database were included. The upper numbers represent the position of variable sites (bp); dots (.) represent a base identical to the sequence from *C. orbiculatus* (HB0695MT01); horizontal line (−) represents a base indel; green border indicates SNP in *C. orbiculatus;* and red border indicate SNPs in *T. wilfordii*.

The sequence analysis showed that there was no site variation among the 11 ITS2 sequences from *C. orbiculatus* (Figure 2). These 11 sequences all had the same length (223 bp), the GC content was 66.4% (Table 1) and the K2P of intraspecific distances was zero (Figure 2). Therefore, we suggest that the major ITS2 haplotype from *C. orbiculatus* is included in the established database. Similar findings were presented for other closely related species including *C. tonkinensis*, *C. angulatus*, *C. hypoleucus,* and *C. vaniotii*. The length of the nine ITS2 sequences from *T. wilfordii* ranged from 221 bp to 223 bp and the GC contents was 59.6–60.2%. There was one variable site (51 bp) in the ITS2 region that divided ITS2 into two sequence haplotypes (Figure 3). One of the haplotypes contains seven sequences, indicating it may be the major ITS2 haplotype of *T. wilfordii*. The range of K2P intraspecific distances among the sequences is from zero to 0.0046 (Figure 2).

The minimum interspecific distance (0.0234) was larger than the maximum intraspecific distance of *C. orbiculatus* (0). Additionally, the significant difference between *C. orbiculatus* and *T. wilfordii* indicated that *C. orbiculatus* can easily be distinguished from *T. wilfordii* and closely related species based on the ITS2 sequences (Figure 2).

High variation is necessary in barcoding to provide accurate taxonomic identification [18]. We analyzed the single nucleotide polymorphisms (SNP) of the ITS2 region among *C. orbiculatus*, *T. wilfordii,* and other closely related species. A total of 62 SNPs were detected in the established database, of which one SNP (133 bp: T) was distinctive for *C. orbiculatus* and nine SNPs (38 bp: T; 46 bp: A; 48 bp: T; 49 bp: T; 107 bp: A; 122 bp: T; 152 bp: A; 176 bp: A; 222 bp: C) were distinctive for *T. wilfordii*. Thus, although there was a high similarity among the ITS2 sequences of *Celastrus* species and *T. wilfordii*, these SNPs can still effectively discriminate *C. orbiculatus* from other *Celastrus* species and *T. wilfordii* (Figure 3).

Furthermore, a neighbor-joining (NJ) tree was constructed (Supplementary Figure S1), which illustrates the separate clustering of *C. orbiculatus* and *T. wilfordii*. Three species of *Euonymus* L. and one species of *Glyptopetalum* L. were chosen as outgroups based on a previous report [24,25,26]. Other *Celastrus* species also clustered into separate clades. *Celastrus rosthornianus* and *C. rosthornianus* var. *loeseneri* clustered into the same clade, likely because of their very close relationship, which might in itself merit further taxonomic investigations.

According to the DNA barcoding standard operating procedure, the results indicated that using ITS2 as a DNA barcode can effectively distinguish *C. orbiculatus* and its congeners, as well between *C. orbiculatus* and *T. wilfordii*.

### 2.3. Examination of the Species Authenticity for the Herbal Products of C. orbiculatus and T. wilfordii Using ITS2 Barcoding

In this study, 47 herbal materials labeled as Nst, which is *C. orbiculatus* (YC0001YP01–YC0001YP21) or Lgt, which is *T. wilfordii* (YC0002YP01–YC0002YP26) were collected from medicinal herbal markets from seven Chinese provinces (Table S2). The results showed that sequence length ranged from 223 bp to 229 bp for Nst, and 223 bp to 224bp for Lgt. There were 96 variable sites in NST and 25 variable sites in LGT (Supplementary Figures S2 and S3). The degree of sequence variation among the herbal products was greater than the intraspecific divergence of *C. orbiculatus* and *T. wilfordii*, indicating that the commercial herbal samples of *C. orbiculatus* (Nst) and *T. wilfordii* (Lgt) are all not derived from a single species.

**Figure 4:**
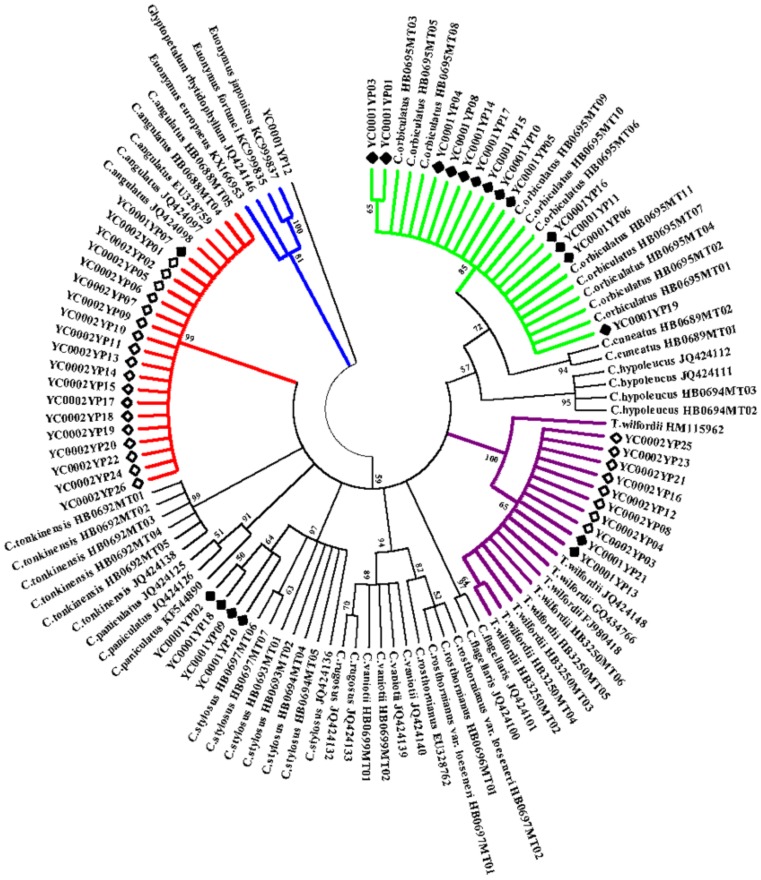
Phylogenetic tree of 59 known species and 47 herbal products available from drug markets using the NJ method. Bootstrap scores (1000 replicates) are shown (≥50%) for each branch. Green shows the clade of *C. orbiculatus*, purple shows the clade of *T. wilfordii*, red shows the clade of *C. angulatus*, and blue shows the clade of outgroups. The solid rhombus represents the products labeled as Nst, which is *Celastrus orbiculatus* and the hollow rhombus represents Lgt, which is *Tripterygium wilfordii*.

We evaluated the composition of the 47 herbal samples and confirmed the best-hit species based on the DNA barcoding (Table S2). A neighbor-joining (NJ) tree was constructed combining the 47 herbal products and the above-mentioned 59 plant samples from the database (Figure 4). We found that some herbal materials were potentially mislabeled. Specifically, 11 of the 21 samples sold as Nst shared 100% homology with *C. orbiculatus* and two of the 21 samples shared 99.6% homology with *C. orbiculatus* (Table S2), and all had the same SNP site as *C. orbiculatus* (Figure S4). The NJ tree showed that 13 samples sold as Nst products and *C. orbiculatus* clustered into one clade (Figure 4). Thus, these thirteen products can be authenticated as *C. orbiculatus*. One Nst products and 18 Lgt products shared 100% identity, and the same SNPs as *C. angulatus* (Figure S4) and clustered with *C. angulatus*. Thus these 19 products were identified as *C. angulatus*. Two Nst products (YC0001YP13 and YC001YP21) and eight Lgt products shared 100% similarity with *T. wilfordii*. These 10 products contained all nine SNPs in *T. wilfordii*, clustered with *T. wilfordii*, thus were identified as *T. wilfordii*. In addition, four Nst products (YC0001YP02, YC0001YP09, YC0001YP18, and YC001YP20) were likely *C. hirsutus* for the high sequence similarity (99.6–100%). One Nst product (YC001YP12) may be derived from Fabaceae, as it shared 100% sequence similarity with *Caesalpinia minax*. In summary, 19 products (40% of the total samples) were derived from *C. angulatus*, 13 (28%) from *C. orbiculatus*, 10 (21%) from *T. wilfordii,* and five from congeneric and other species (Figure S5). ITS2 can be successfully used to distinguish the herbal products of *C. orbiculatus* and *T. wilfordii* available from herbal markets in China.

## 3. Discussion

### 3.1. The ITS2 Barcode Can be Used to Identify Celastrus Species and T. wilfordii 

*Celastrus* L. belongs to Celastraceae, including nearly 30 species throughout the world, and 24 of these species (16 endemic species) are widely distributed in most provinces of China, especially in south of the Yangtze river [6,27]. However, the classification of *Celastrus* is ambiguous for variable morphology and polymorphic traits. Recently, various molecular techniques have started to facilitate the study of Celastraceae [24,25,28,29,30,31]. Mu et al. used ETS (expressed sequence tag), ITS and three plastid DNA loci (psbA-trnH, rpl16, and trnL-F) for the first time to assess the phylogenetic relationship within the *Celastrus* L. [26]. The ITS2 locus, a relatively short segment on the ITS region, has been proposed to use as a universal DNA barcode for the identification of medicinal plants and their closely related species [19,32]. Here, we publish the first use of the ITS2 barcode to distinguish *Celastrus* species and *T. wilfordii*. In our ITS2 database, we downloaded 21 reference ITS2 sequences from GenBank, including 17 sequences for *Celastrus* L. and four sequences for *Tripterygum wilfordii*. NJ tree showed that the sequences submitted in this study and the ones from GenBank could clustered into one clade, respectively. This indicated the accuracy of morphological identification and the power of DNA barcoding in plant taxonomy [18,19]. There is rich variation in ITS2 region, and the average interspecific divergence is higher than the average intraspecific divergence within almost all of the included *Celastrus* species and *T. wilfordii*. The only exception is *C. rosthornianus var. loeseneri* and *C. rosthornianus*, which were not distinguished from one another in the NJ tree or by genetic distance. Our results highlight the advantages of using the universal marker ITS2 as a DNA barcode for medicinal plants [18,19,33].

### 3.2. DNA Barcoding Can be Used to Trace Herbal Products of C. orbiculatus and T. wilfordii in Markets 

The accurate identification of herbal materials is closely related to the safety of clinical medication. Both *C. orbiculatus* and *T. wilfordii* have been used for the treatment of RA, but the species have very different toxicity levels and side effects in humans. Therefore, the accurate identification of *C. orbiculatus* and *T. wilfordii* is necessary. In this study, all 59 sequences of known species were included in a database, which was used to authenticate 47 herbal products bought from herbal markets in China. Our results indicate that the herbal products of *C. orbiculatus* and *T. wilfordii* collected from the markets are often misidentified. In fact, only 28% of samples sold as *C. orbiculatus* (Nst) were authenticated as *C. orbiculatus*, and 21% were authenticated as *T. wilfordii*. The remaining samples were adulterants. Among the adulterants, two samples of the 21 sold as Nst were identified using ITS2 as *T. wilfordii*. Over 50% of the market products labeled as Lgt (*T. wilfordii*) were found to be adulterated by *C. angulatus*, which is in line with results from previous research [29,34]. Based on the NJ tree, both Nst and Lgt are adulterants for each other, which highlights a potential safety issue. In addition, *Caesalpinia minax* was a potential adulterant that clustered separately in the NJ tree. Its seeds, which is called ‘ku-shi-lian’ in Guangxi (China), have long been used as folk medicine for the treatment of common cold, influenza, fever, and dysentery [35]. ‘Nan-she-le’, another conventional name for *C. minax* that similar to Nan-she-teng (Nst), may be responsible for the confusion [36]. *Celastrus hirsutus* and *Caesalpinia minax* were not included in our ITS2 database, However, it wouldn’t hinder the examination of the species authenticity for the herbal products of *C. orbiculatus* and *T. wilfordii*, which emphasizes the applicability of our method. Our study showed that DNA barcoding was capable of quickly and effectively distinguishing herbal products from adulterants, and confirmed the traceability of ITS2. We found numerous examples of safety issues among the herbal products of *C. orbiculatus* and *T. wilfordii* available in herb markets, and the mislabeling of herbal products is of concern. However, there are still challenges for designing specific ITS2 markers to identify adulterations in the herbal products of *C. orbiculatus* and *T. wilfordii* without sequencing. In this study, only one SNP (133 bp: T) was distinctive for *C. orbiculatus* and there was a high similarity among the ITS2 sequences of other *Celastrus* species (Figure 2). It is difficult to find the primers with enough variation. Attempts are necessary to expand the original plant database, and screening of more supplementary DNA loci to develop specific barcodes for medicinal material identification. In addition, due to the progress of sequencing technology and lower cost, direct DNA sequencing is recommended. It is an accurate, reliable, rapid, more cost-effective and robust tool, especially for the short molecular marker. The produced sequences can be used to conveniently detect and trace the adulteration of traditional medical products.

### 3.3. The Application of DNA Barcoding for Traceability 

The adulteration of herbal materials is a serious threat for the safety and effectiveness of clinical drugs. All plant materials, including seeds, seedlings, harvested materials, processing, and more, need to be inspected for quality and authenticated, and this process needs to be carefully documented. Herbgenomic and functional genomes have provided abundant genetic resources for the development of molecular markers of medicinal herbs [37,38,39,40], including amplified fragment length polymorphism (AFLP), simple repeat sequence interval (ISSR), single nucleotide polymorphisms (SNP) for genome, and simple sequence repeat (SSR) in transcriptome [41,42,43,44]. DNA barcode is one of the most highly concerning molecular markers [18,19]. DNA barcoding technology is expected to fulfill this goal by allowing the tracing of raw materials starting from the source through all steps in the processing chain. At present, Chen (2010) and colleagues have established the largest DNA barcode system of identification for herbal materials and constructed an online TCM (TCMD) DNA barcode database [19], a DNA barcoding platform for identifying herbal materials for professionals and the public (http://www.tcmbarcode.cn). It also provides a method and platform for users to perform species identification using DNA barcoding. The methods for DNA barcoding of herbal materials have been approved for incorporation into Supplement 3 of the Chinese Pharmacopoeia (2010 edition). Researchers believe that DNA barcoding represents a renaissance for the identification of herbal medicine [18]. It is also an effective technology for tracing the origin of raw materials and detecting adulterants in herbal medicine sold at markets. Verification of the accuracy and stability of ITS2 as the DNA barcode continues beyond herbal materials [33,45,46], and has been applied to Pulsatillae Radix (*Pulsatilla chinensis*) [47], Rhodiola Crenulatae Radix et Rhizoma (*Rhodiola Crenulatae*) [45], Notopterygii Rhizoma et Radix (*Notopterygium incisum*) [48], Menispermi Rhizoma (*Menispermum dauricum*) [49], and Lonicerae Japonica Flos (*Lonicerae Japonicae*) [50]. DNA barcoding has also been used to trace the composition of the health food [15,45,46]. For these reasons, we used ITS2 to trace the herbal products of *C. orbiculatus* and *T. wilfordii* in market circulation. In this study, all sequences of original plant materials were submitted into the TCMD as a database and all detected samples from the market could be matched with this database. One sample matched with Fabaceae with high similarity, which also emphasizes the coverage of our database (Table S2).

The DNA barcoding identification system of herbal materials currently contains 78,847 sequences belonging to 23,262 species, and more than 95% of the crude herbal drugs published in the domestic and international pharmacopoeia. This technology may not confirm the definite name of all adulterants, but it does have the ability to confirm adulterants and can be informative for distinguishing authentic materials from adulterants. With the increase of species and population coverage in this system, we believe it can authenticate results and be used for accurate identification. Moreover, DNA barcoding in conjunction with the database can be applied to herbal products with no background information, and the continued advances in sequencing technology can increasingly authenticate the herbal ingredients of herbal medicine. Thus, we predict that DNA barcoding will play a stronger role in the traceability of traditional Chinese medicinal materials in the future.

## 4. Materials and Methods 

### 4.1. Plant and Herbal Materials

Thirty-eight samples were collected from Enshi and Shennongjia in Hubei Province and Lushan Mountain in Jiangxi Province (Table S1). All corresponding voucher samples were deposited at the College of Pharmacy, Hubei University of Chinese Medicine, Wuhan, Hubei Province, China. In addition, we included 21 published ITS2 sequences downloaded from GenBank (Table S1). Other than the voucher samples, 47 herbal products labeled as Nst and Lgt were collected from larger medicinal markets in seven Chinese provinces (Table S3). Using the DNA barcoding technology, we tested the species composition of the herbal materials available in the markets.

### 4.2. DNA Extraction

The sample’s surface was first wiped with 75% ethanol, and then cut into small pieces (40–50 mg). Silica-gel dried leaves (25 mg) or roots (40–50 mg) were pulverized for two minutes at a frequency of 30 times/second in Mixer Mill MM400 (Retsch GmbH, Haan, Germany). The extraction of total genomic DNA was performed from the ground materials using the modified CTAB method, following a previously described protocol [51]. Each pulverized sample was mixed with 800 μL cetyltrimethylammonium bromide (CTAB) extraction buffer (preheated at 65 °С) in a 2 mL sterile reaction tube. The mixture was incubated at 65 °С for 40 min and gently shaken well every 10 min. Further, 750 μL chloroform isoamylol (volume ratio is 24:1) was added, vortexed vigorously and centrifuged at 12,000× *g* for 5 min. The supernatant (750 μL) was transferred to a new 2 μL sterile reaction tube, mixed with 750 μL of chloroform isoamylol, vortexed vigorously and centrifuged at 12,000× *g* for 5 min. This step was repeated and the main function is to extract and remove protein in samples. After that, the upper phase (600 μL) was mixed with equal volume of isopropyl alcohol (pre-cooling at −20 °С) in a 1.5 mL sterile reaction tube. After incubation for 30 min at −20 °С, the tube was incubated for 20–30 min at room temperature. The mixture was then transferred to a silicon matrix column (spin columns CB3, Cat. No. RK130, TianGen, Beijing, China) and centrifuged at 12,000× *g* for 1 min. The liquid in the collection tube of column was discarded and the column was washed with 70% ethanol (700 μL). After incubation for 5 min at room temperature and centrifugation at 12,000× *g* for 1 min, the liquid in the collection tube of column was discarded. This step was repeated. After centrifugation at 12,000× *g* for 2 min, the column was transferred to a new 1.5 μL sterile reaction tube and air-dried at room temperature for 20 min. 40–80 μL deionized water (preheated at 65 °С) was added on the column, incubated for 5 min at room temperature and centrifuged at 12,000× *g* for 2 min. The total DNA was stored at −20 °С. The quality and quantity of total DNA were accessed and determined by a bioanalyzer (Agilent Technologies, Santa Clara, CA, USA) and agarose gel electrophoresis. The DNA quality was meet for the subsequent amplification and sequencing. Our results highlighted the universality of this method for use on diverse materials including roots, fruits, stems, leaves and flowers [33,50,52,53].

### 4.3. Amplification, Sequencing, and Sequence Analysis

General PCR reaction conditions and procedures for total DNA amplification have been previously described [19,23]. Briefly, PCR reactions consisted of a total of 25 μL containing 12.5 μL of 2× PCR Mix, 2 μL (2.5 μM) of both ITS2-P3 (5′-YGACTCTCGGCAACGGATA-3′) and ITS2-E4 (5′-RGTTTCTTTCCTCCGCTTA-3′) primers, 2 μL DNA template and 8.5 μL ddH2O. All of the steps were performed according to DNA barcoding standard operating procedures (DNA barcoding SOP) [19]. Agarose gel electrophoresis (AGE) was used to visualize PCR products; 95 PCR products displayed a single band. The PCR products were successfully sequenced, and high-quality bidirectional sequences were obtained. CondonCode Aligner V 4.2.4 (CondonCodeCo, USA.) was used to proofread and assemble trace sequencing files. Using the HMMer annotation method and removing the 5.8S and 28S sections at both ends of the sequences based on the Hidden Markov model (HMM), the complete ITS2 region was obtained [54]. MEGA (Molecular Evolutionary Genetics Analysis) 6.0 was used to align sequences [55], and then the intraspecific and interspecific K2P genetic distances were calculated and used to construct a Neighbor-Joining (NJ) tree [56].

## Figures and Tables

**Table 1:** Sequence characteristics of the ITS2 (internal transcribed spacer) barcode database for *Celastrus* and *Tripterygium*.

Latin Name	No. of Sequences	Sequence Length (bp)	GC Content (%)	Haplotype No.	No. of Intraspecific Variation Sites
*Celastrus orbiculatus*	11	223	66.4	1	0
*C. stylosus*	7	222–223	68.0–68.6	2	1
*C. tonkinensis*	6	221	66.9	1	0
*C. angulatus*	5	223	66.8	1	0
*C. hypoleucus*	4	221	66.5	1	0
*C. vaniotii*	4	223	66.8	1	0
*C. cuneatus*	2	222	66.2	1	0
*C. rosthornianus*	2	223	68.1–68.6	2	1
*C. rosthornianus* var. *loeseneri*	2	223	67.7–68.1	2	1
*C. paniculatus*	3	223	66.3–67.3	3	2
*C. rugosus*	2	231	66.7	1	0
*C. flagellaris*	2	230	66.9–67.4	2	4
*Tripterygium wilfordii*	9	221–223	59.6–60.2	2	1
Total	59	221–231	59.6–68.6	-	-

## Supplementary Materials

Supplementary materials are available on line.
