# Supplementary figures and images for: Investigation on Species Authenticity for Herbal Products of Celastrus Orbiculatus and Tripterygum Wilfordii from Markets Using ITS2 Barcoding
Source: Molecules. 2018 Apr 21;23(4):967. doi: 10.3390/molecules23040967 (PMC6017776; doi:10.3390/molecules23040967)

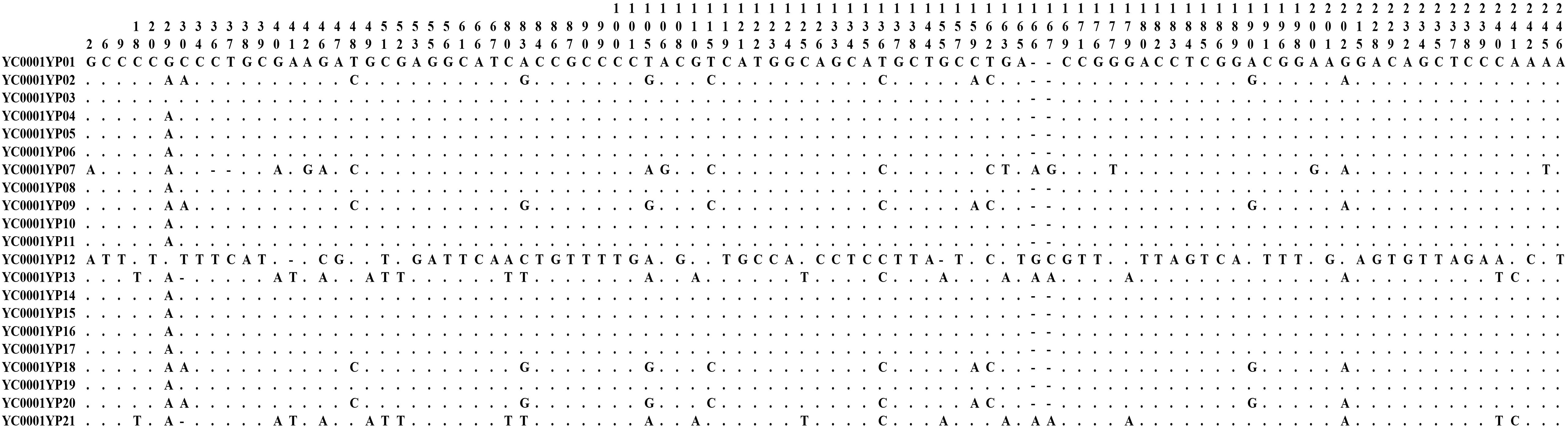

Supplement: Supplementary file 1 [file molecules-23-00967-s001.zip › supplementary mateiral/Supplementary Figure S2.tif]

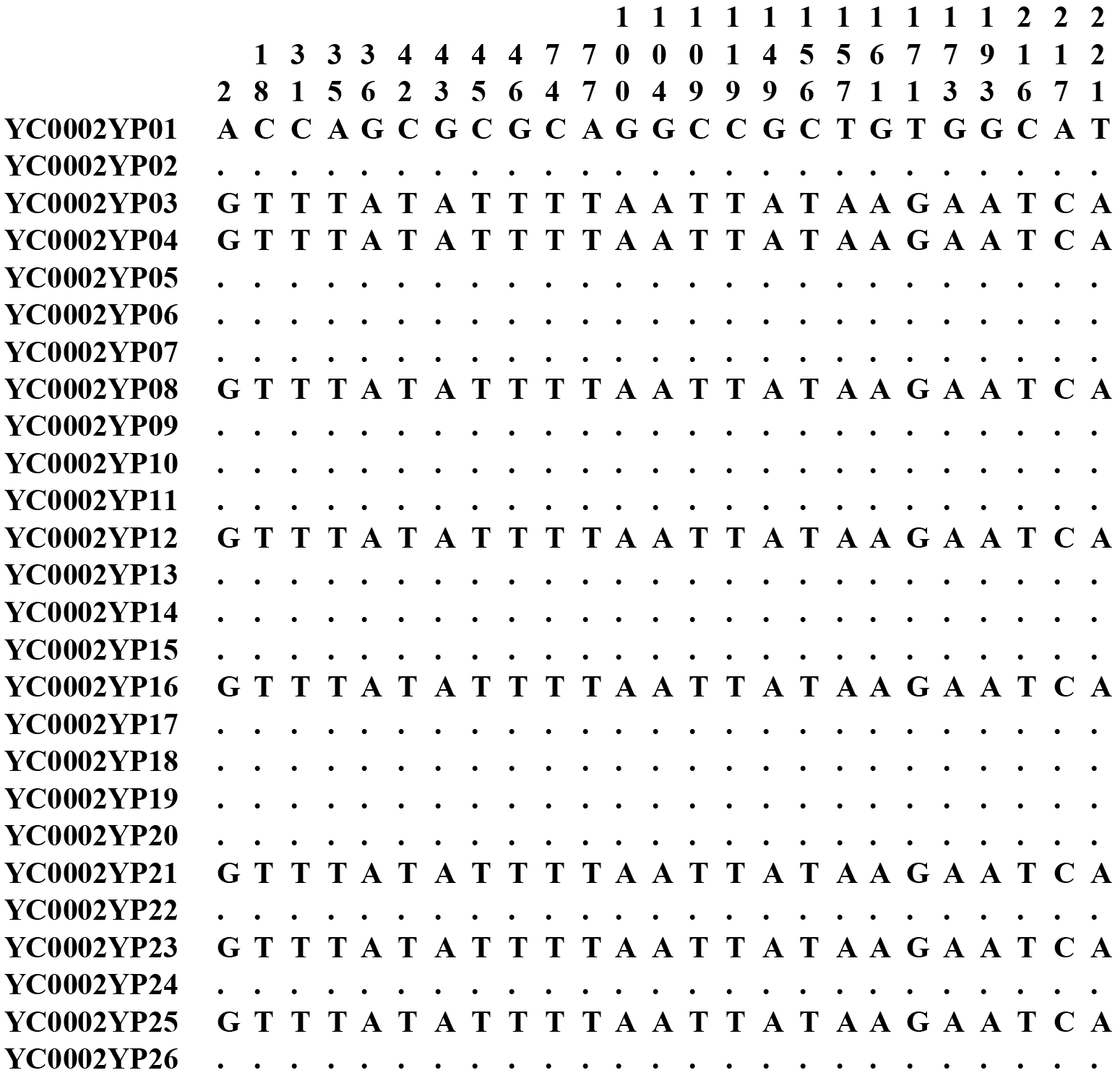

Supplement: Supplementary file 1 [file molecules-23-00967-s001.zip › supplementary mateiral/Supplementary Figure S3.tif]

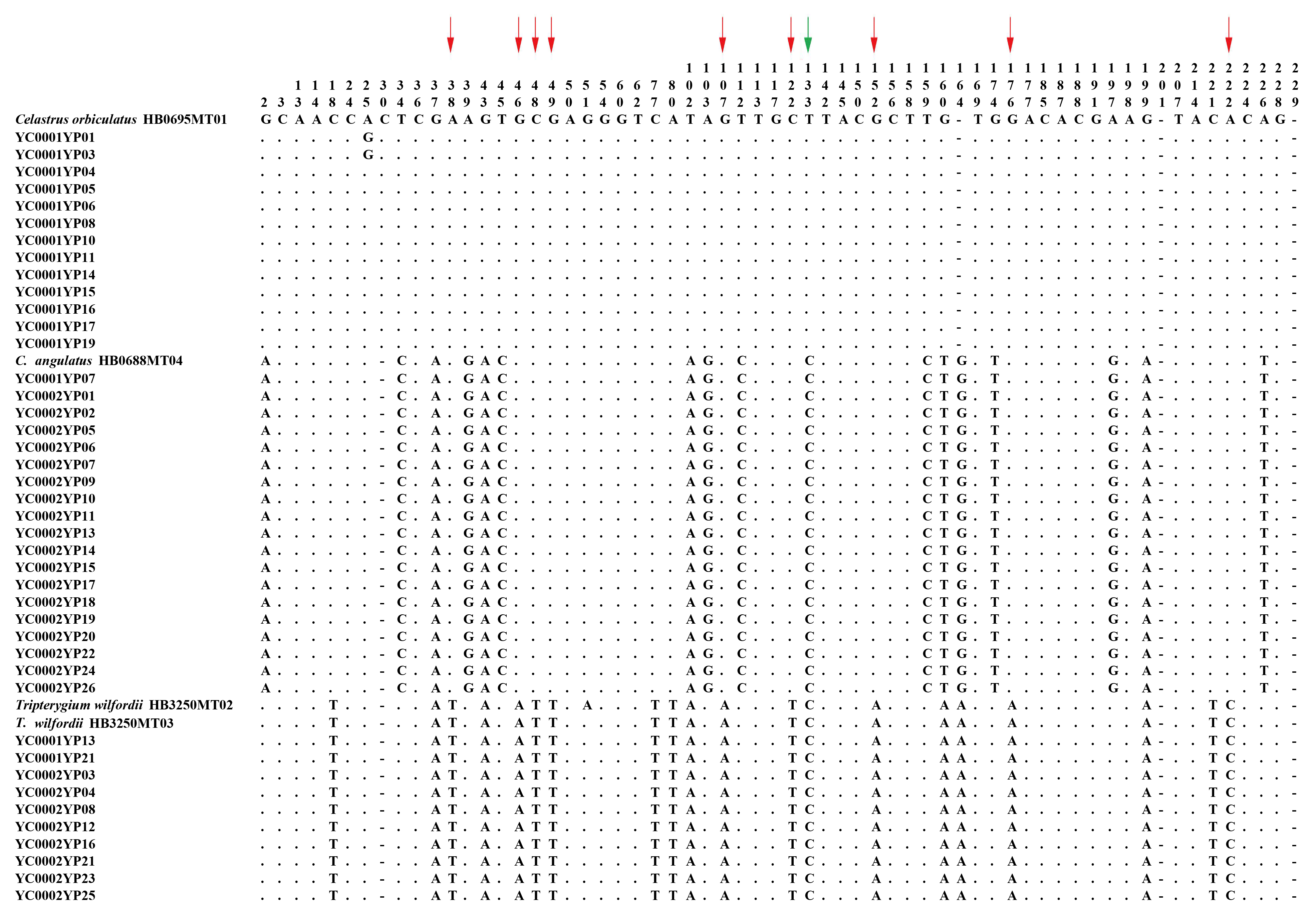

Supplement: Supplementary file 1 [file molecules-23-00967-s001.zip › supplementary mateiral/Supplementary Figure S4.tif]

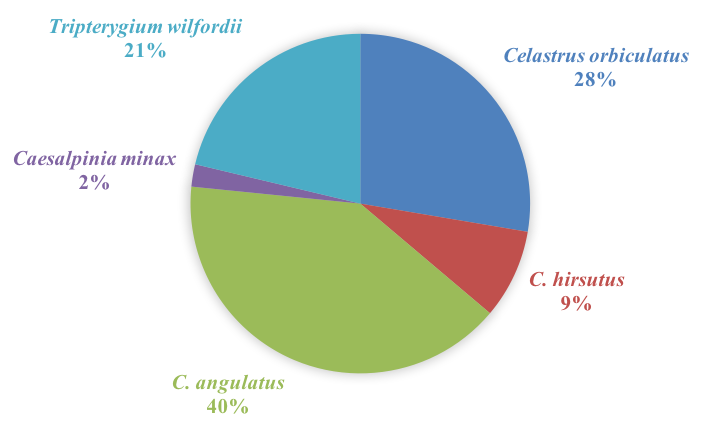

Supplement: Supplementary file 1 [file molecules-23-00967-s001.zip › supplementary mateiral/Supplementary Figure S5.tif]

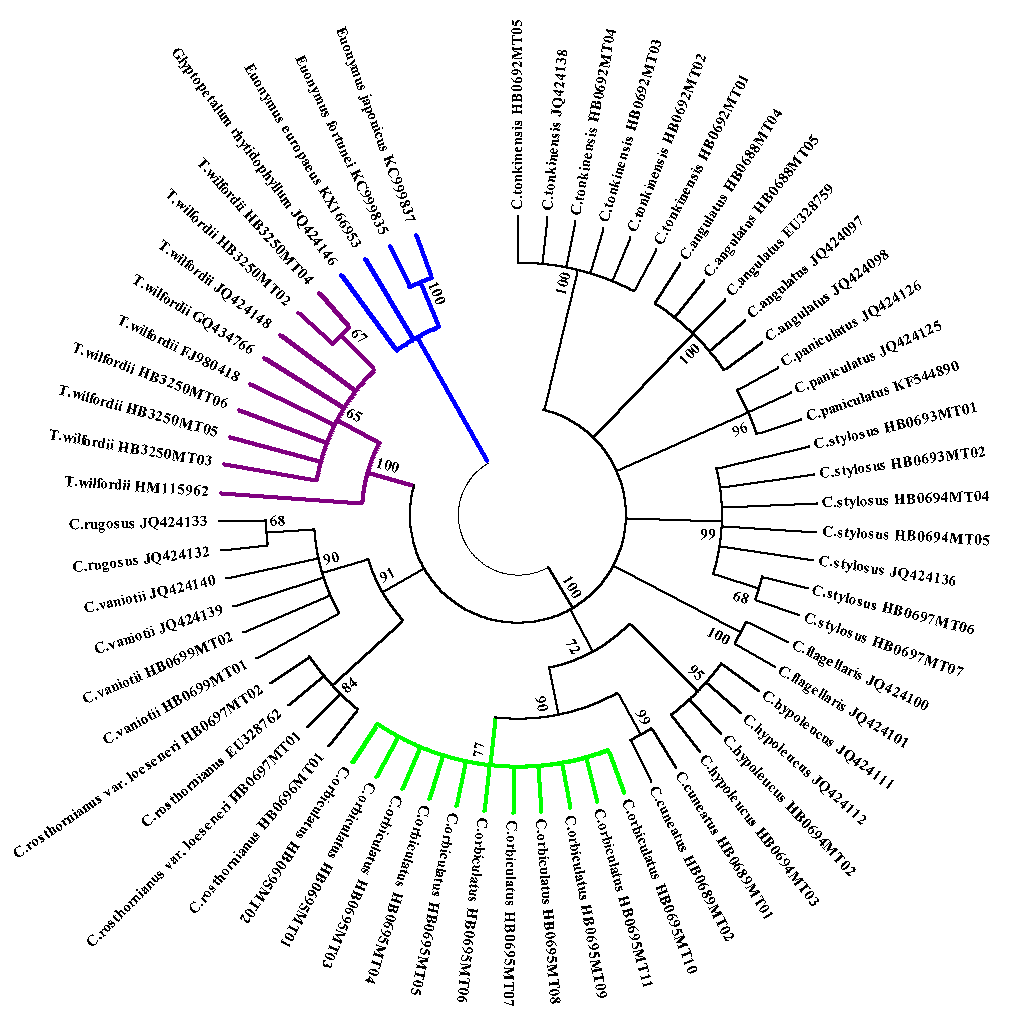

Supplement: Supplementary file 1 [file molecules-23-00967-s001.zip › supplementary mateiral/Supplementary Figure S1.png]
