# Supplementary material for: Investigation on Species Authenticity for Herbal Products of Celastrus Orbiculatus and Tripterygum Wilfordii from Markets Using ITS2 Barcoding
Source: Molecules. 2018 Apr 21;23(4):967. doi: 10.3390/molecules23040967 (PMC6017776; doi:10.3390/molecules23040967)
Supplement: Supplementary file 1 [file molecules-23-00967-s001.zip › supplementary mateiral/Supplementary Table S1.doc]

Supplementary Table S1 List of specimens and downloaded sequences for *Celastrus* and *Tripterygium*.

| Latin name | No. of samples | Gen Bank No. | Voucher No. | Source location  (In China) | Downloaded sequence No. from Gen Bank |
| --- | --- | --- | --- | --- | --- |
| *Celastrus orbiculatus* | 11 | KX431087–097 | HB0695MT01–11 | Enshi,Hubei  Shennongjia,Hubei  Lushan,Mountain | - |
| *Celastrus stylosus* | 6 | KX431103–108 | HB0693MT01–02,  HB0694MT04–05,  HB0697MT06–07, | Enshi,Hubei | 1(JQ424136) |
| *Celastrus tonkinensis* | 5 | KX431098–102 | HB0692MT01–05 | Enshi,Hubei  Shennongjia,Hubei | 1(JQ424138) |
| *Celastrus angulatus* | 2 | KX431083–084 | HB0688MT04–05 | Shennongjia,Hubei | 3(EU328759;  JQ424097–98) |
| *Celastrus hypoleucus* | 2 | KX431081–082 | HB0694MT02–03 | Shennongjia,Hubei | 2(JQ424111–12) |
| *Celastrus vaniotii* | 2 | KX431111–112 | HB0699MT01–02 | Enshi,Hubei | 2(JQ424139–140) |
| *Celastrus cuneatus* | 2 | KX431109–110 | HB0689MT01–02 | Enshi,Hubei | - |
| *Celastrus rosthornianus* | 1 | KX431080 | HB0696MT01 | Enshi,Hubei | 1(EU328762) |
| *Celastrus rosthornianus* var. *loeseneri* | 2 | KX431085–086 | HB0697MT01–02 | Enshi,Hubei | - |
| *Celastrus paniculatus* | - |  | - | - | 3(JQ424125–126;  KF544890) |
| *Celastrus rugosus* | - |  | - | - | 2(JQ424132–133) |
| *Celastrus flagellaris* | - |  | - | - | 2(JQ424100–101) |
| *Tripterygium wilfordii* | 5 | KX431113–117 | HB325MT02–06 | Enshi,Hubei | 4(FJ980418; GQ434766;  HM115962; JQ424148) |
| Total | 38 |  |  |  | 21 |

Symbol “-” refers to the samples which do not have the relevant information.
