# Supplementary material for: Investigation on Species Authenticity for Herbal Products of Celastrus Orbiculatus and Tripterygum Wilfordii from Markets Using ITS2 Barcoding
Source: Molecules. 2018 Apr 21;23(4):967. doi: 10.3390/molecules23040967 (PMC6017776; doi:10.3390/molecules23040967)
Supplement: Supplementary file 1 [file molecules-23-00967-s001.zip › supplementary mateiral/Supplementary Table S2.doc]

Supplementary Table S3 Herbal decoction piece samples sold as “Nan-she-teng” and “Lei-gong-teng” from different Chinese provinces.

| Label Name | Voucher No. | Purchased Place (In China) | Sequence length (bp) | best-hit species | Similarity / % |
| --- | --- | --- | --- | --- | --- |
| Nansheteng | YC0001YP01 | Hubei | 223 | *Celastrus orbiculatus* | 99.6% |
| Nansheteng | YC0001YP02 | Jilin | 224 | *Celastrus hirsutus* | 99.6% |
| Nansheteng | YC0001YP03 | Bozhou,Anhui | 223 | *Celastrus orbiculatus* | 99.6% |
| Nansheteng | YC0001YP04 | Liaoning | 223 | *Celastrus orbiculatus* | 100% |
| Nansheteng | YC0001YP05 | Hubei | 223 | *Celastrus orbiculatus* | 100% |
| Nansheteng | YC0001YP06 | Hubei | 223 | *Celastrus orbiculatus* | 100% |
| Nansheteng | YC0001YP07 | Sichuan | 223 | *Celastrus angulatus* | 100% |
| Nansheteng | YC0001YP08 | Sichuan | 223 | *Celastrus orbiculatus* | 100% |
| Nansheteng | YC0001YP09 | Yizhou,Guangxi | 223 | *Celastrus hirsutus* | 100% |
| Nansheteng | YC0001YP10 | Jilin | 223 | *Celastrus orbiculatus* | 100% |
| Nansheteng | YC0001YP11 | Yunnan | 223 | *Celastrus orbiculatus* | 100% |
| Nansheteng | YC0001YP12 | Liaoning | 229 | *Caesalpinia minax* | 100% |
| Nansheteng | YC0001YP13 | Shennongjia,Hubei | 223 | *Tripterygium wilfordii* | 100% |
| Nansheteng | YC0001YP14 | Bozhou,Anhui | 223 | *Celastrus orbiculatus* | 100% |
| Nansheteng | YC0001YP15 | Bozhou,Anhui | 223 | *Celastrus orbiculatus* | 100% |
| Nansheteng | YC0001YP16 | Jilin | 223 | *Celastrus orbiculatus* | 100% |
| Nansheteng | YC0001YP17 | Hubei | 223 | *Celastrus orbiculatus* | 100% |
| Nansheteng | YC0001YP18 | Sichuan | 224 | *Celastrus hirsutus* | 100% |
| Nansheteng | YC0001YP19 | Sichuan | 223 | *Celastrus orbiculatus* | 100% |
| Nansheteng | YC0001YP20 | Yizhou,Guangxi | 223 | *Celastrus hirsutus* | 100% |
| Nansheteng | YC0001YP21 | Shennongjia,Hubei | 223 | *Tripterygium wilfordii* | 100% |
| Leigongteng | YC0002YP01 | Bozhou,Anhui | 223 | *Celastrus angulatus* | 100% |
| Leigongteng | YC0002YP02 | Bozhou,Anhui | 224 | *Celastrus angulatus* | 100% |
| Leigongteng | YC0002YP03 | Bozhou,Anhui | 223 | *Tripterygium wilfordii* | 100% |
| Leigongteng | YC0002YP04 | Hubei | 223 | *Tripterygium wilfordii* | 100% |
| Leigongteng | YC0002YP05 | Bozhou,Anhui | 223 | *Celastrus angulatus* | 100% |
| Leigongteng | YC0002YP06 | Guizhou | 223 | *Celastrus angulatus* | 100% |
| Leigongteng | YC0002YP07 | Guizhou | 224 | *Celastrus angulatus* | 100% |
| Leigongteng | YC0002YP08 | Kunming,Yunnan | 223 | *Tripterygium wilfordii* | 100% |
| Leigongteng | YC0002YP09 | Hubei | 223 | *Celastrus angulatus* | 100% |
| Leigongteng | YC0002YP10 | Bozhou,Anhui | 223 | *Celastrus angulatus* | 100% |
| Leigongteng | YC0002YP11 | Baoding,Hebei | 223 | *Celastrus angulatus* | 100% |
| Leigongteng | YC0002YP12 | Yulin,Guangxi | 224 | *Tripterygium wilfordii* | 100% |
| Leigongteng | YC0002YP13 | Baoding,Hebei | 223 | *Celastrus angulatus* | 100% |
| Leigongteng | YC0002YP14 | Baoding,Hebei | 223 | *Celastrus angulatus* | 100% |
| Leigongteng | YC0002YP15 | Baoding,Hebei | 223 | *Celastrus angulatus* | 100% |
| Leigongteng | YC0002YP16 | Dali,Yunnan | 223 | *Tripterygium wilfordii* | 100% |
| Leigongteng | YC0002YP17 | Guizhou | 224 | *Celastrus angulatus* | 100% |
| Leigongteng | YC0002YP18 | Guizhou | 223 | *Celastrus angulatus* | 100% |
| Leigongteng | YC0002YP19 | Bozhou,Anhui | 223 | *Celastrus angulatus* | 100% |
| Leigongteng | YC0002YP20 | Bozhou,Anhui | 223 | *Celastrus angulatus* | 100% |
| Leigongteng | YC0002YP21 | Bozhou,Anhui | 224 | *Tripterygium wilfordii* | 100% |
| Leigongteng | YC0002YP22 | Bozhou,Anhui | 223 | *Celastrus angulatus* | 100% |
| Leigongteng | YC0002YP23 | Kunming,Yunnan | 224 | *Tripterygium wilfordii* | 100% |
| Leigongteng | YC0002YP24 | Huibei | 223 | *Celastrus angulatus* | 100% |
| Leigongteng | YC0002YP25 | Yulin,Guangxi | 223 | *Tripterygium wilfordii* | 100% |
| Leigongteng | YC0002YP26 | Baoding,Hebei | 223 | *Celastrus angulatus* | 100% |
